# Association of *TNFRSF1B* Promoter Polymorphisms with Human Disease: Further Studies Examining T-Regulatory Cells Are Required

**DOI:** 10.3389/fimmu.2018.00443

**Published:** 2018-03-06

**Authors:** Hongchuan Li, Stephen K. Anderson

**Affiliations:** ^1^Basic Science Program, Leidos Biomedical Research Inc., Frederick National Laboratory for Cancer Research, Frederick, MD, United States

**Keywords:** tumor necrosis factor, TNFR2, T-regulatory cells, promoter, variable number tandem repeat, transcription

## Abstract

The TNFR2 receptor is expressed by highly active regulatory T cells, and thus constitutes an important therapeutic target for the treatment of autoimmune disease and cancer. Disease susceptibility as well as the potential response to therapies directed at TNFR2 could be significantly impacted by genetic variation in the promoter of the *TNFRSF1B* gene that codes for the TNFR2 protein. To date, only a few studies have examined the association of *TNFRSF1B* promoter variation with disease, and the potential impact on T-regulatory cell (Treg) number and function has not been examined. We propose that copy number variation of a key transcription factor binding site has a significant effect on *TNFRSF1B* promoter activity, and should be considered in studies of disease susceptibility and especially with regard to variation in the level of TNFR2 expression on Tregs.

## Introduction

Tumor necrosis factor (TNF) is a multifunctional cytokine that can affect multiple cellular responses, such as inflammation, tumorigenesis, viral replication, septic shock, and autoimmunity (1). These functions are mediated through binding of TNF to either the TNFR1 (*TNFRSF1A* gene) or TNFR2 (*TNFRSF1B* gene) receptors. TNFR1 is expressed by most cells, and likely accounts for the pleiotropic effects of TNF administration, including the severe side effects associated with systemic administration (2). TNFR2 expression is more restricted, with significant levels expressed by a subset of highly suppressive T-regulatory cells (Tregs) in both mouse and human (3, 4). TNFR2 has also been detected in the central nervous system (5), on cardiac myocytes (6), and on thymocytes (7). TNF signaling through TNFR1 has been associated with apoptosis, while TNFR2 receptor stimulation generally leads to a proliferative response (8). Accordingly, TNFR1 and TNFR2 have differences in their signaling pathways, although there is some overlap (9). TNF binding to TNFR1 triggers apoptosis through the activation of the TNFR1-associated death domain and Fas-associated death domain adaptor proteins. By contrast, TNFR2 signaling uses TRAF2, leading to NF-κB activation, resulting in enhanced growth and survival (10). However, IL-2 stimulation of T cells induces both TNFR2 and RIP expression that results in apoptotic cell death in response to TNFR2 signaling (11). The more restricted expression of TNFR2 makes it a more attractive molecular target for drug development than TNFR1. TNFR2-expressing Tregs are abundant in human and murine tumors (12), and TNFR2 is also expressed by multiple tumors and promotes their growth (13). It therefore appears that TNFR2 can play an important role in tumor development by suppressing the immune response in addition to promoting tumor cell growth. A recent study has demonstrated that an antagonistic anti-TNFR2 antibody was capable of inhibiting Treg proliferation and directly killed the OVCAR3 ovarian cancer cell line that has high levels of TNFR2 expression, suggesting that targeting TNFR2 may be an effective anti-tumor therapy (14).

## Association of TNFR2 Alterations with Disease

The level of TNFR2 signaling would be expected to have a significant effect on the proliferation of T cells. A recent study of TNFR2-deficient effector T cells (Teffs) demonstrated the importance of this receptor for the proliferative expansion of Teffs (15). Whole exome sequencing of patients with cutaneous T cell lymphomas (Mycosis fungoides and Sézary syndrome) revealed that 38% had alterations that would positively effect TNFR2 signaling (16). 14% of patients demonstrated a copy number gain of the *TNFRSF1B* gene that was correlated with increased *TNFRSF1B* mRNA levels and increased TNFR2 protein in a cell line with increased gene copy number. 4% of patients had a mutation at codon 377 of TNFR2 that enhanced NF-κB signaling.

Genome-wide association studies have implicated the *TNFRSF1B* gene in two human diseases, systemic lupus erythematosus [SLE (17)] and anti-neutrophil cytoplasmic antibody (ANCA) in inflammatory bowel disease (18). In SLE, a M196R variant was associated with the disease: however, the functional relevance of this change was not determined. In ANCA, a SNP in the first intron was associated with decreased TNFR2 levels in carriers of the SNP associated with susceptibility, but the mechanism behind the decrease in TNFR2 was not investigated.

Although changes in TNFR2 levels were associated with disease susceptibility, none of the studies cited above looked directly at variation within the *TNFRSF1B* promoter region. A 15 bp insertion/deletion has been identified within the core promoter region that affects the copy number of a repeated 15 bp sequence (19), and it is referred to as a variable number tandem repeat (VNTR). The repeated sequence contains a predicted SP1-binding site, and therefore the number of repeats might have an effect on promoter activity. Several studies have examined the effect of variation in the promoter VNTR on disease (20–22). A study of patients with hematologic malignancies treated with prolonged chemotherapy (20) showed that the susceptibility to develop invasive pulmonary aspergillosis (IPA) was decreased in individuals that were homozygous for *TNFRSF1B* alleles containing two copies of the repeat (2/2 genotype). 43% of the patients that developed IPA had the 2/2 genotype, whereas 69% of patients without IPA were 2/2 (*p* = 0.03). In a study of bone mineral density, individuals that were homozygous for a single copy of the repeat (1/1 genotype) had a lower rate of lumbar spine bone loss than subjects that had at least one allele with two copies of the element (21). Individuals possessing either the 2/2 or 1/2 genotype had bone loss of 0.84%, whereas individuals with the 1/1 genotype lost 0.39% (*p* = 0.017). In lupus (SLE) patients, patients with the 2/2 genotype had reduced disease activity, as measured by reduced renal involvement and higher C3 levels (22). 71% of patients with the 1/1 genotype were renal disorder positive, as compared to 29% for the 1/2 or 27% for the 2/2 genotypes (*p* = 0.05). The increased copy number of an element with a putative SP1-binding site could result in a higher level of *TNFRSF1B* transcription, however, none of these studies examined the level of TNFR2 expression.

## Functional Effect of Copy Number Variation of An SP1-Binding Element in *TNFRSF1B*

**Figure 1:**
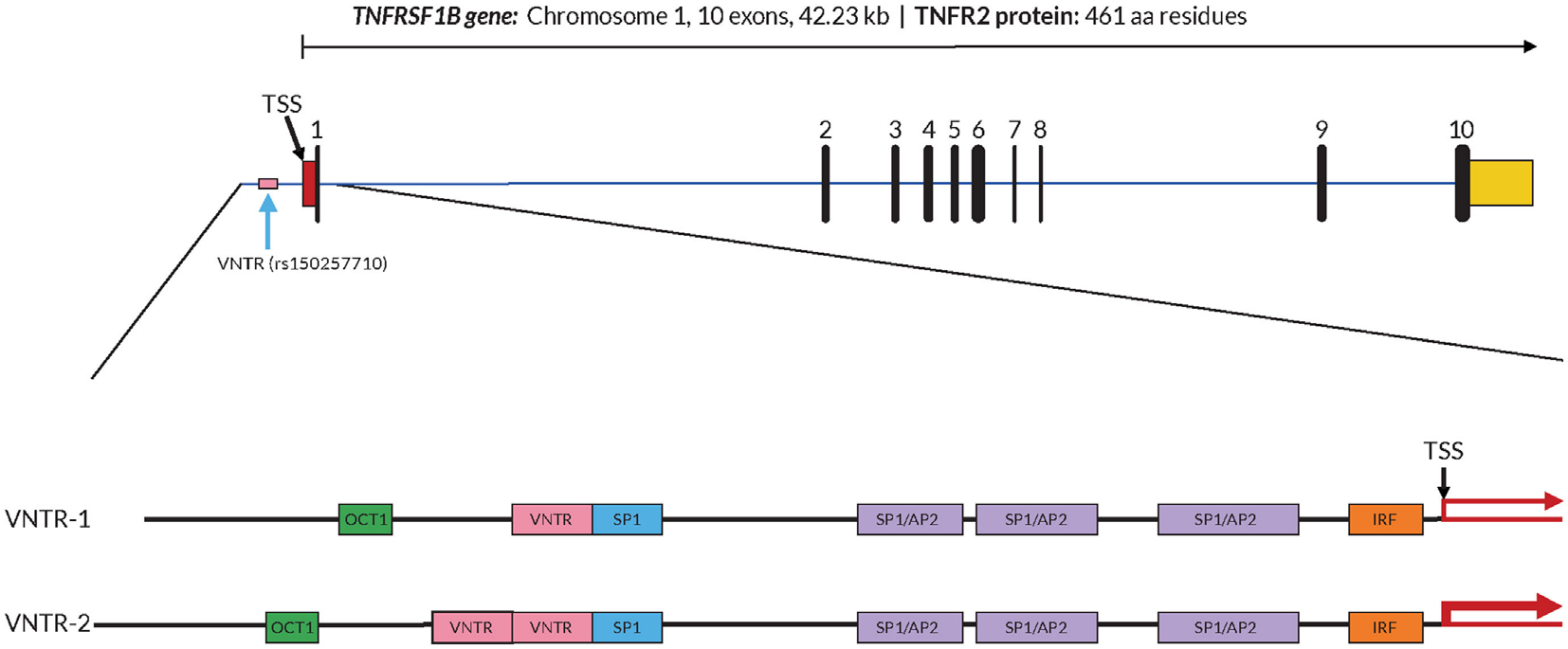
Structure of the *TNFRSF1B* gene and core promoter region. A schematic representation of the exon–intron organization of the human *TNFRSF1B* gene. The exons are indicated by the numbered black rectangles. The 5′-untranslated and 3′-untranslated regions are indicated by red boxes and yellow boxes, respectively. The position of the variable number tandem repeat (VNTR) element is shown, and the dbSNP reference number is indicated in parentheses. The 419 bp 5′-flanking region of the human proximal *TNFRSF1B* promoter analyzed is shown in an expanded view, with the positions of putative transcription factor binding sites shown as colored boxes. Promoter variants with either 1 (VNTR-1) or 2 (VNTR-2) copies of the VNTR are shown. The transcription start site (TSS) is marked with a black arrow, and the relative levels of transcription are indicated by red arrows of differing thickness.

**Figure 2:**
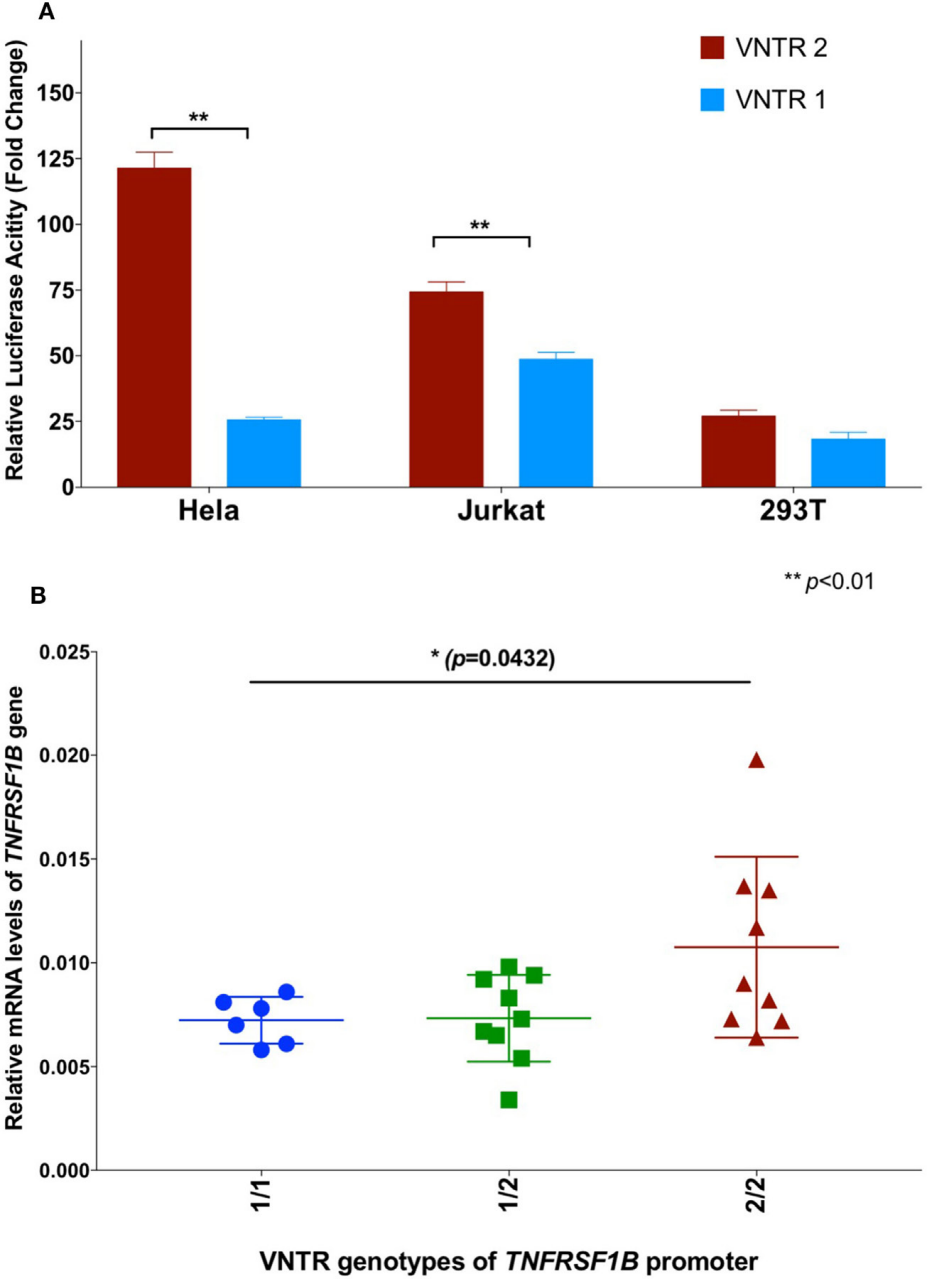
**(A)** Effect of variable number tandem repeat (VNTR) number on *TNFRSF1B* promoter activity. Activity of pGL3-luciferase reporter constructs containing the genomic sequence of the *TNFRSF1B* gene from −475 to −57 relative to the start codon, were transfected into Hela, Jurkat and 293T cells. Promoter fragments with either two copies of the VNTR (VNTR-2) or one copy (VNTR-1) were compared. The average fold activity of constructs relative to empty pGL3 vector from at least three independent experiments is shown. Error bars represent ±1 SEM. An unpaired *t*-test with Welch’s correction was used to calculate statistical significance. **(B)** QPCR of *TNFRSF1B* transcripts. Total cellular RNA was purified from peripheral blood mononuclear cells isolated from healthy donors (NCI-Frederick Research Donor Program; http://ncifrederick.cancer.gov/programs/science/rdp/default.aspx) and cDNA synthesis was carried out using random hexamer primer. Real time RT-PCR primers were: *TNFRSF1B* exon1-For 5′-CTGGGCTGCGGCGCACGCCTTG-3′; TNFRSF1B exon2-Rev 5′-GCAGCACATCTGAGCTGTCTGG-3′. HPRT1-For 5′-TGAGGATTTGGAAAGGGTGT-3′; HPRT1-Rev 5′- GAGCACACAGAGGGCTACAA-3′. Relative mRNA levels of *TNFRSF1B* were normalized to HPRT1 by the delta CT method. ANOVA was used to calculate statistical significance.

In order to further investigate a possible functional effect of the copy number variation on promoter activity, we performed an *in vitro* analysis of the core *TNFRSF1B* promoter region. Figure 1 shows a schematic of the *TNFRSF1B* gene and the identification of potential transcription factor binding sites. The two copies of the perfect 15 base pair (bp) repeat that constitutes the VNTR are shown, followed by a partial repeat containing the first 10 bp of the repeat, but including a predicted SP1-binding site that is also present in the VNTR. The 419 bp region starting 386 bp upstream of the transcription start site (TSS) to 33 bp downstream of the TSS was cloned into the pGL3 luciferase reporter vector, and the relative luciferase activity of promoter fragments containing either one or two copies of the element was determined. As shown in Figure 2A, two copies of the repeat had a significantly higher promoter activity than 1 copy in HeLa cells and the Jurkat T cell line. The 293 cell line showed a small effect, but this was not statistically significant. This suggests that individuals bearing two copies of the 15 bp repeat should have a higher rate of transcription of the *TNFRSF1B* gene, and increased expression of TNFR2. The predicted effect of VNTR copy number on transcription was tested by comparing *TNFRSF1B* transcript levels in peripheral blood lymphocytes from donors with either the 1/1, 1/2, or 2/2 VNTR genotypes (Figure 2B). Donors with the 2/2 genotype had a significantly higher level of *TNFRSF1B* transcript than donors with the 1/1 genotype.

## Conclusion and Perspective

Although genetic variation in the TNFR2 receptor has been associated with several human diseases, we believe that additional studies examining variation in the copy number of the VNTR bearing a predicted SP1-binding site in the *TNFRSF1B* promoter region are warranted. We have shown that there is a direct functional effect on promoter activity and transcript levels, which would be predicted to affect receptor levels. Several studies have associated changes in TNFR2 levels with susceptibility to disease, so one would expect the VNTR to also show association in these diseases. Previous studies that have used exon sequencing would of course have missed the effect of this genetic variation. Given that the frequency of *TNFRSF1B* alleles lacking one of the repeats is in the range 20–30% depending on the population studied, there is likely substantial variation in TNFR2 levels due to the VNTR that may be associated with susceptibility to multiple diseases. The majority of previous work on the genetic association of *TNFRSF1B* variation with disease was performed before the importance of this receptor in Treg function was appreciated (3, 4). The effect of *TNFRSF1B* gene variation on the number and function of Tregs represents an avenue of research that could shed considerable light on the mechanisms behind the disease associations observed. If the *TNFRSF1B* gene variant containing two copies of the VNTR leads to increased expression of TNFR2 on Treg cells, this could result in increased Treg cell numbers and activity. Higher Treg levels could potentially explain the observation that lupus patients with two copies of the VNTR had reduced disease activity (22).

In addition to the predicted role of variation in TNFR2 expression on Treg function and the control of autoimmunity, there may also be significant effects on the response of T effector cells in cancer patients treated with checkpoint inhibitors. If TNFR2-directed reagents are eventually introduced into the clinic, it may be informative to look for associations between the response to such agents, and the VNTR status of the individual.

## Author Contributions

SA performed data analysis and wrote the manuscript. HL performed experiments, analyzed data, and wrote the manuscript.

## Disclaimer

The content of this publication does not necessarily reflect the views or policies of the Department of Health and Human Services, nor does mention of trade names, commercial products or organizations imply endorsement by the US Government.

## Conflict of Interest Statement

SKA and HL are employed by Leidos Biomedical Research Incorporated, the Operations and Technical Support contractor for the National Cancer Institute.
